# Factors affecting the accuracy of prehospital triage application and prehospital scene time in simulated mass casualty incidents

**DOI:** 10.1186/s13049-024-01257-3

**Published:** 2024-09-26

**Authors:** Luca Carenzo, Lorenzo Gamberini, Federico Crimaldi, Davide Colombo, Pier Luigi Ingrassia, Luca Ragazzoni, Francesco Della Corte, Marta Caviglia

**Affiliations:** 1 Department of Anesthesia and Intensive Care Medicine, IRCCS Humanitas Research Hospital, Humanitas Clinical and Research Center – IRCCS, Via Manzoni 56, 20089 Rozzano, MI Italy; 2 Department of Anesthesia, Intensive Care and Prehospital Emergency, Maggiore Hospital Carlo Alberto Pizzardi, Bologna, Italy; 3 Department of Anesthesia and Critical Care, Ospedale “Ss. Trinità”, Borgomanero, Italy; 4 CRIMEDIM - Center for Research and Training in Disaster Medicine, Humanitarian Aid and Global Health, Università del Piemonte Orientale, Novara, Italy; 5 Centro Professionale Sociosanitario, Centro di Simulazione (CeSi), Lugano, Switzerland; 6 Department for Sustainable Development and Ecological Transition, Università del Piemonte Orientale, Vercelli, Italy; 7 Department of Translational Medicine, Università del Piemonte Orientale, Novara, Italy

## Abstract

**Background:**

The contemporary management of mass casualty incidents (MCIs) relies on the effective application of predetermined, dedicated response plans based on current best evidence. Currently, there is limited evidence regarding the factors influencing the accuracy of first responders (FRs) in applying the START protocol and the associated prehospital times during the response to MCIs. The objective of this study was to investigate factors affecting FRs’ accuracy in performing prehospital triage in a series of simulated mass casualty exercises. Secondly, we assessed factors affecting triage-to-scene exit time in the same series of exercises.

**Methods:**

This retrospective study focused on simulated casualties in a series of simulated MCIs Full Scale Exercises. START triage was the triage method of choice. For each Full-Scale Exercise (FSEx), collected data included exercise and casualty-related information, simulated casualty vital parameters, simulated casualty anatomic lesions, scenario management times, and responder experience.

**Results:**

Among the 1090 casualties included in the primary analysis, 912 (83.6%) were correctly triaged, 137 (12.6%) were overtriaged, and 41 (3.7%) were undertriaged. The multinomial regression model indicated that increasing heart rate (RRR = 1.012, *p* = 0.008), H-AIS (RRR = 1.532, *p* < 0.001), and thorax AIS (T-AIS) (RRR = 1.344, *p* = 0.007), and lower ISS (RRR = 0.957, *p* = 0.042) were independently associated with overtriage. Undertriage was significantly associated with increasing systolic blood pressure (RRR = 1.013, *p* = 0.005), AVPU class (RRR = 3.104 per class increase), and A-AIS (RRR = 1.290, *p* = 0.035). The model investigating the factors associated with triage-to-scene departure time showed that the assigned prehospital triage code red (TR = 0.841, *p* = 0.002), expert providers (TR = 0.909, *p* = 0.015), and higher peripheral oxygen saturation (TR = 0.998, *p* < 0.001) were associated with a reduction in triage-to-scene departure time. Conversely, increasing ISS was associated with a longer triage-to-scene departure time (TR = 1.004, 0.017).

**Conclusions:**

Understanding the predictors influencing triage and scene management decision-making by healthcare professionals responding to a mass casualty may facilitate the development of tailored training pathways regarding mass casualty triage and scene management.

**Supplementary Information:**

The online version contains supplementary material available at 10.1186/s13049-024-01257-3.

## Background

Contemporary management of mass casualty incidents (MCIs) relies on the effective application of predetermined dedicated response plans based on current best evidence. Quick decision-making, efficient triage, and reduced scene time are essential in MCIs to optimize medical response efforts, mitigate the strain on available resources, and improve overall outcomes for those affected [1].

MCI Triage involves the systematic classification of patients based on predefined algorithms, allowing prioritization for treatment and onward transfer, and best use of available resources both on-scene and in-hospital [2, 3]. The accuracy of the triage process, is gauged by how well the assigned priority aligns with the expected priority, strictly adhering to a predefined algorithm [4]. If there’s a discrepancy between the assigned and expected priorities, it can lead to two types of errors: overtriage and undertriage. These errors can significantly impact the efficiency and effectiveness of patient care in both ordinary times and MCIs [5, 6]. Undertriage and overtriage represent undesirable outcomes, as they can lead to suboptimal allocation of resources and potentially negative consequences for patients [7, 8]. A commonly applied system for MCI triage is the START system which involves categorizing individuals with color-coded tags: green (minor injury), yellow (delayed), red (immediate), and black (deceased). Key parameters evaluated within this system are the ability to walk, breathing and its rate, capillary refill time, and the ability to follow commands [7]. Accurate triage should be coupled to smooth scene management, and it is known that prolonged prehospital intervals are linked to increased in-hospital mortality [9, 10]. Empirical evidence from real-world data indicates that MCIs tend to lead to longer time intervals in the prehospital phase compared to non-MCIs [11, 12].

Currently, there is limited evidence regarding the factors influencing the accuracy of first responders (FRs) in applying the START protocol and the associated prehospital times during response to MCIs. One contributing aspect to this limitation is the need for more reliable real-world data to assess such factors. Full-scale exercises (FSEx) serve as the most common approximation to real events, incorporating realistic scenarios, high-fidelity simulated casualties, and actual resources (ambulances, personnel, hospital beds, etc.) in real-time. FSEx not only represent the gold standard in training for most EMS and hospital systems but also offer a valuable opportunity to gather data that could allow for the identification of factors impacting MCI triage and prehospital times, thus enabling professionals involved in MCI management and training to develop targeted interventions and protocols that effectively address the current challenges [13–17]. Therefore, after evaluating the accuracy related to the application of both prehospital and hospital triage within our cohort, the principal objective of this study was to investigate factors affecting FRs’ accuracy in performing prehospital triage in a series of simulated mass casualty exercises. Secondly, we assessed factors affecting triage-to-exit time in the same series of exercises.

## Methods

This retrospective observational study encompassed all consecutive FSEx conducted by the Center for Research and Training in Disaster Medicine, Humanitarian Aid, and Global Health (CRIMEDIM; Università del Piemonte Orientale; Novara, Italy) within its institutional activity over ten years (from January 2012 to December 2022).

### Exercise design

All FSEx were designed and executed in a uniform and standardized manner. Each incorporated a storyboard that outlined a predetermined number of casualties. The severity and distribution of these casualties were established based on epidemiological reports, with the objective to closely resemble the characteristics of the targeted event. Once epidemiological profiles were defined, specific sets of simulated casualties were generated accordingly. Casualty sets comprised three components: (i) paper Dynamic Casualty Cards (DCCs), which featured evolving vital signs and were designed to be placed into transparent plastic envelopes and safely attached to a lanyard to be worn around the casualty neck; (ii) instructions for casualties ‘moulage’, providing professional guidance on creating a standard and high-fidelity wounds on the actors for a realistic appearance and (iii) storyboards for simulating realistic and evolving patients, serving as guidelines to match the data cards and simulate injuries and associated symptoms. All simulated casualties underwent standardized training on how to progress according to the provided storyboard, passing time and receiving treatments. The instructions given to participants in delivering treatments to simulated victims were: (i) to place the necessary treatment devices (e.g., fluids, medications) near the victim; (ii) to communicate the administration of the treatment; (iii) to remain in close proximity to the victim for a duration realistically corresponding to the treatment time. DCCs featured all necessary vitals required for assigning triage codes based on the START system. Based on these vitals, each casualty had a predetermined expected (correct) triage code. In addition to their role as casualties, participants also collected data by documenting their assigned triage codes at the scene and upon arrival at the hospital, as well as key timestamps during the exercise, including time to triage, time to a prehospital staging area, and time to prehospital scene exit. All these times were measured from the start of the exercise. These data are then computed and used in the after-action debriefing. Details about the casualty evolution method, general structure of the simulation, and DCCs were described in a series of previous papers. [16, 18–24] An example of DCCs is presented in Additional file 1: Fig. 1S.

**Fig. 1:**
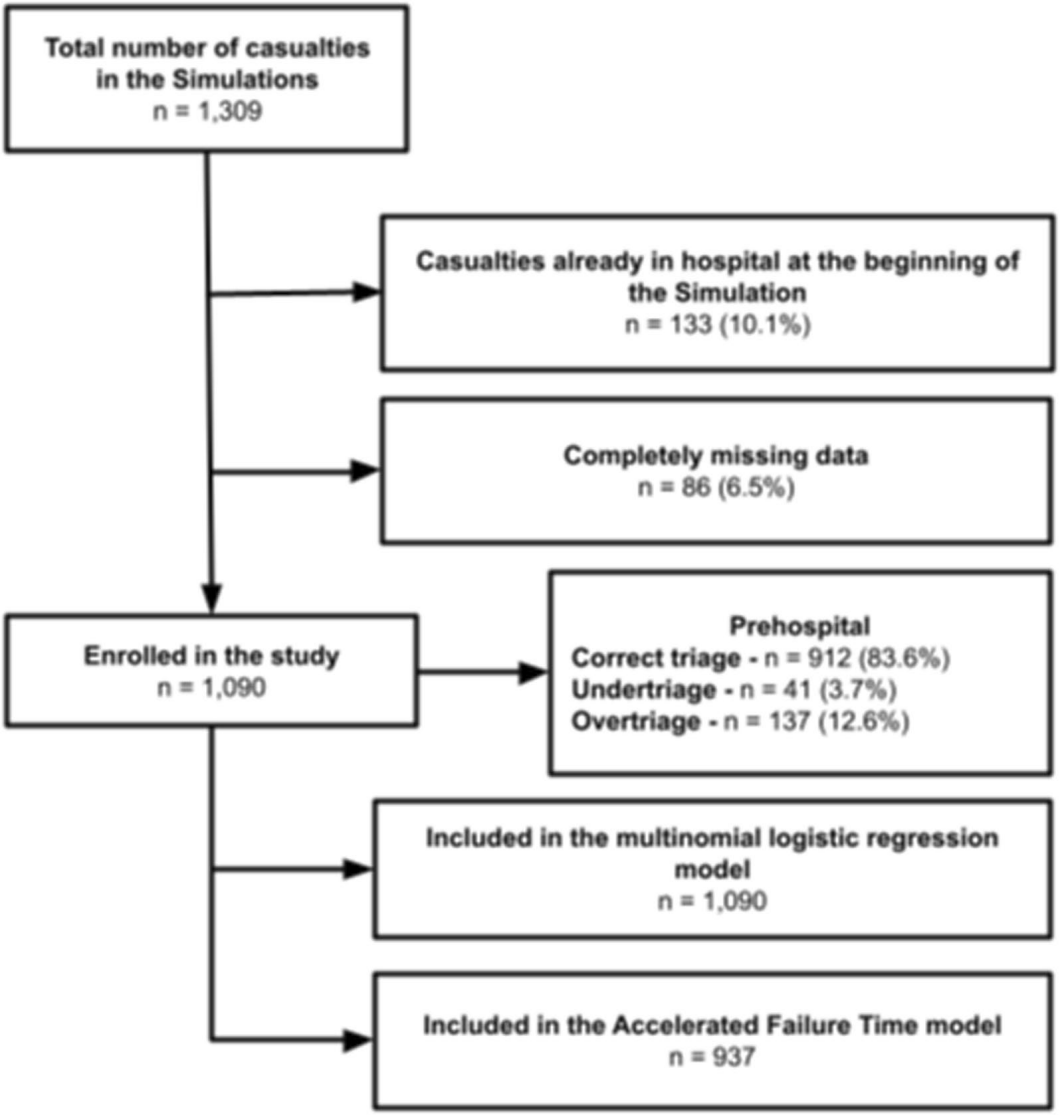
Flowchart of simulated casualties in the study

### Inclusion and exclusion criteria

To be enrolled in the study, each FSEx had to incorporate at least one prehospital scenario and at least one simulated hospital. The analysis excluded all casualties specifically designated to be observed only at the hospital and did not undergo prehospital triage.

### Data collection

For each FSEx, the master database including pre-existing exercise-specific casualty profiles, expected and assigned triage codes, and key management times, was thoroughly reviewed and consolidated into a single file for analysis.

Included variables consisted of:

- Exercise and casualty-related data—exercise unique identification number, simulated casualty unique identification number, expected prehospital first triage following the START algorithm, assigned prehospital and hospital triage.
- DCC simulated vital parameters—heart rate, systolic blood pressure, blood oxygen saturation, level of consciousness (AVPU scale)
- Simulated anatomical injuries—pre-defined casualty injury severity score (ISS), pre-defined abbreviated injury scales (AIS).
- Scenario management times—time-to-triage, time to Collecting Area, Time from Triage to Scene Departure, Prehospital Scene Time, Time to Hospital, Time to final disposition.
- Experience of the group of rescuers—Exercises managed by trained professionals completing a Master of Science in Disaster Medicine (European master in disaster medicine, EMDM,) [25] were arbitrarily classified as “expert”, while exercises managed by junior doctors or trainees who completed a basic course in disaster medicine were classified as “non-expert”. This classification is based on our previous work [16].

### Statistical analysis

Casualties were grouped into three categories: correct triage, overtriage, and undertriage, based on how the assigned triage matched the expected one.

Continuous variables were represented as either medians and interquartile ranges (IQR) or means and standard deviations, depending on their distribution assessed with QQ plots. The Kruskal–Wallis test or one-way analysis of variance was used to compare these variables as needed. Categorical variables were presented as counts and percentages, and the Chi-square test or Fisher’s exact test was used for comparison when suitable.

A multinomial logistic regression model was constructed to examine the impact of various physiological and anatomical factors on the risk of overtriage or undertriage in prehospital settings. In this model, triage evaluation was the dependent variable, with correct triage serving as the reference outcome. The effects of individual factors under investigation were expressed as relative risk ratios (RRR).

Vital parameters, Injury Severity Score (ISS), Abbreviated Injury Scale (AIS) scores, and the experience of the operators were tested as independent variables in the model. A stepwise forward and backward selection of variables was performed based on the Akaike Information Criterion (AIC).

For the secondary objective, the time from triage to exit was used. This metric was considered less influenced by the spatial distribution of casualties within the scenario and the rescue teams’ exploration path. A survival analysis approach was adopted to investigate factors influencing the triage-to-exit time.

The full model, which included anatomical lesions, physiological parameters, the group’s experience, and observed triage as independent covariates, fit a Weibull distribution well (Additional file 1: Fig. 2S). However, the assumption of proportional hazards was not met. As a result, an accelerated failure time (AFT) model was constructed. Variable selection for this model was also performed using a stepwise forward and backward selection based on AIC. AFT models are parametric survival models that distribute the probability of failure over time by accelerating or decelerating it among groups. The output estimator for each covariate in these models is the time ratio (TR), which measures how much longer or shorter the time-to-event is on average. The main advantage of AFT models is that they do not rely on the assumption of proportional hazards. All the tests were two-tailed, a *p* < 0.05 was considered significant. The analyses were performed with R Core Team 2023 (R: A Language and Environment for Statistical Computing. R Foundation for Statistical Computing, Vienna, Austria).

**Fig. 2:**
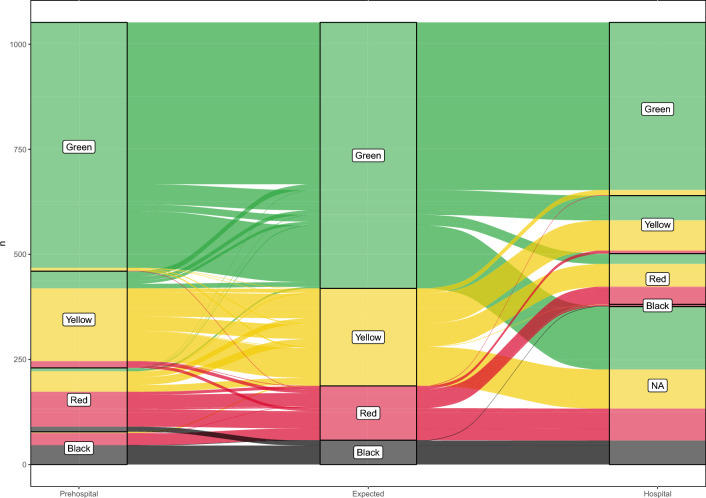
Alluvial plot portraying the casualty triage in the study exercise. *Note*: this alluvial plot visually depicts casualty triage during the study exercise. It shows expected and assigned triage codes at prehospital and in-hospital stages, with stream widths representing casualty numbers. Colours denote triage codes: Green, Yellow, Red, Black, and NA (for unadmitted patients)

### Ethics and data protection

Ethical approval was not required as the study focused solely on documenting even frequencies within simulated training programs for learning improvement, with no supplementary interventions conducted. All the authors confirm adhering to the principles outlined in the Declaration of Helsinki while delivering the simulated educational program, ensuring confidentiality. Information stored in the master databases was fully anonymized and was recorded at a group level, due to the inability to associate a particular individual with a specific visited simulator.

## Results

The study incorporated data from 13 FSEx conducted between 2012 and 2022, encompassing 1309 simulated casualties. A comprehensive description of each FSEx included in the study can be found in the electronic supplement (Additional file 1: Table 1S).

**Table 1:** Descriptive Characteristics of study casualties stratified by correctness of assigned triage

	Correct triage	Overtriage	Undertriage	*p*
n	912	137	41	
Heart Rate (median [IQR]), bpm	92 [84, 110]	101 [88, 120]	110 [0, 130]	0.002
Respiratory Rate (median [IQR]), bpm	22 [18, 25]	23 [19, 26]	23 [0, 32]	0.071
Systolic Blood Pressure (median [IQR]), mmHg	120 [100, 132]	110 [90, 130]	100 [0, 132]	0.041
Diastolic Blood Pressure (median [IQR]), mmHg	76 [55, 85]	70 [51, 80]	68 [0, 88]	0.012
SpO_2_ (median [IQR]), %	97 [92, 98]	94 [85, 97]	92 [0, 95]	< 0.001
ISS (median [IQR])	4 [1, 5]	4 [1, 16]	10 [1, 75]	< 0.001
Time to Triage (median [IQR]), min	41 [28, 62]	54 [39, 80]	34 [26, 51]	< 0.001
Time from Triage to Scene Departure (median [IQR]), min	75 [47, 103]	75 [49, 93]	68 [44, 106]	0.903
Prehospital Scene Time (median [IQR]), min	120 [91, 151]	127 [105, 168]	111 [74, 156]	0.109
Time to Hospital (median [IQR]), min	132 [102, 161]	136 [108, 176]	120 [79, 146]	0.225
Time to final disposition (median [IQR]), min	178 [151, 204]	180 [155, 204]	140 [99, 172]	0.004
Expert, n(%)	667 (73.1)	108 (78.8)	27 (65.9)	0.192

Out of the 1309 simulated casualties, 133 (10.1%) did not undergo prehospital triage as they were planned to be already inside the hospital at the beginning of the simulation. Additionally, 86 simulated casualties (6.5%) had complete missing data. Figure 1 shows the flow of the simulated casualties included in the study and the corresponding relative numbers included in the analysis. Among the 1090 casualties included in the primary analysis, 912 (83.6%) were correctly triaged, 137 (12.6%) were overtriaged, and 41 (3.7%) were undertriaged. Table 1 shows descriptive characteristics of included casualties stratified by the correctness of the assigned prehospital triage, while Fig. 2 shows the expected and assigned triage codes in prehospital and hospital assessments.

**Fig. 3:**
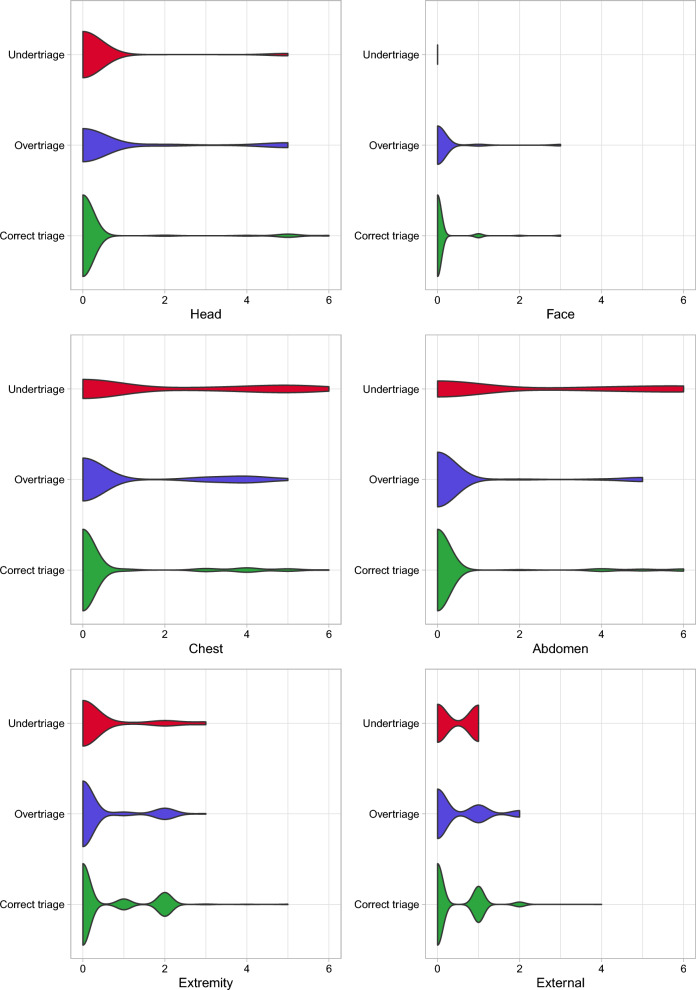
Distribution of regional Abbreviated Injury Scores (AIS) across the various groups. *Note*: this violin plot illustrates the distribution of regional Abbreviated Injury Scores (AIS) across different groups: correct, undertriage, and overtriage. The violin shape represents the density of AIS values, with wider sections indicating higher frequency

Concerning vital parameters, the overtriaged and undertriaged groups exhibited lower blood pressure, peripheral oxygen saturation and level of consciousness (AVPU class); conversely, heart rate and ISS were higher in both these groups compared to the correctly triaged simulants. Figure 3 illustrates the distribution of regional AIS scores across the various groups. Specifically, head, chest and abdomen AIS scores (H-AIS, T-AIS and A-AIS) were significantly higher in mistriaged casualties, while Face AIS scores (F-AIS) were lower. Of note, undertriaged casualties had a shorter time-to-triage, whereas overtriaged casualties experienced a longer time-to-triage. However, no differences were observed in terms of operator experience in the unadjusted population.

The multinomial regression model resulting from stepwise variable selection (Table 2) evidenced that increasing heart rate (RRR = 1.012, *p* = 0.008), H-AIS (RRR = 1.532, *p* < 0.001) and thorax AIS (T-AIS) (RRR = 1.344, *p* = 0.007), and lower ISS (RRR = 0.957, *p* = 0.042) were independently associated with overtriage. On the other hand, undertriage was significantly associated with increasing systolic blood pressure (RRR = 1.013, *p* = 0.005), AVPU class (RRR = 3.104 per class increase), and A-AIS (RRR = 1.290, *p* = 0.035). The AFT model resulting from stepwise variable selection (Table 3) investigating the factors associated with triage-to-scene departure time, showed that the assigned prehospital triage code red (TR = 0.841, *p* = 0.002), expert providers (TR = 0.909, *p* = 0.015) and higher peripheral oxygen saturation (TR = 0.998, *p* < 0.001) were associated with a reduction in triage-to-scene departure time. Conversely increasing ISS was associated with a longer triage-to-scene departure time (TR = 1.004, 0.017).

**Table 2:** Multinomial logistic regression model for overtriage and undertriage

Variable	RRR	95%CI	*p*
*Overtriage*			
Expert group	1.526	0.963–2.418	0.072
Heart rate (per point increase)	1.012	1.003–1.021	0.008
Systolic pressure (per mmHg increase)	1.000	0.996–1.004	0.938
AVPU class (per class increase from A)	1.305	0.969–1.758	0.080
Injury severity score (1 pt increase)	0.957	0.918–0.998	0.042
AIS—Head	1.532	1.217–1.931	< 0.001
AIS—Chest	1.344	1.083–1.667	0.007
AIS—Abdomen	1.273	0.978–1.659	0.073
*Undertriage*			
Expert group	0.529	0.257–1.089	0.084
Heart rate (per point increase)	1.005	0.993–1.017	0.413
Systolic blood pressure (per mmHg increase)	1.013	1.004–1.022	0.005
AVPU class (per class increase from A)	3.104	1.908–5.048	< 0.001
Injury severity score (1 pt increase)	1.005	0.972–1.040	0.754
AIS—Head	0.734	0.507–1.061	0.100
AIS—Chest	0.957	0.733–1.249	0.746
AIS—Abdomen	1.290	1.018–1.634	0.035

Reference group: Correct triage

*RRR* relative risk ratio; *CI* confidence interval; *AIS* Abbreviated Injury scale

**Table 3:** Factors influencing triage-to-exit time

Variable	TR	95%CI	*p*
*Prehospital triage colour assigned*			
Green	Reference		
Yellow	1.062	0.981–1.149	0.139
Red	0.841	0.754–0.940	0.002
Black	0.990	0.780–1.256	0.933
Expert providers group	0.909	0.841–0.982	0.015
Injury severity score (1 point increase)	1.004	1.001–1.007	0.017
SpO2 (1 point increase)	0.998	0.997–0.999	< 0.001
AVPU class (per class increase from A)	0.950	0.886–1.018	0.147

Multivariate Accelerated Failure Time model

*TR* time ratio; *CI* confidence interval; *SpO*_2_ Blood oxygen saturation

Multivariable Accelerated failure time model

## Discussion

This study investigated the influencing factors on FRs’ accuracy in prehospital triage application and triage-to-exit time during simulated mass casualty exercises. It represents a pioneering effort in systematically investigating the impact of these variables, with potential implications for customizing MCI triage training. Our findings indicate an overall FRs accuracy of 83.6%, a higher figure compared to the results reported in the literature [5]. Specifically, we report an overall overtriage rate of 12.6% and a notably lower overall undertriage rate (3,7%). Undertriage refers to the incorrect assignment of lower triage priority to patients with severe injuries or medical conditions, thus causing delay or insufficiency in providing medical care to those who require it with utmost urgency [2]. It can lead to preventable complications, worsening of injuries, and even loss of life [26]. Conversely, overtriage occurs when patients with less severe injuries receive higher triage priority. This can overwhelm emergency medical services and hospitals, resulting in inefficient resource utilization, increased waiting times, and delays in treating patients in need of immediate medical intervention [27]. It is crucial to emphasize that, in our study, triage accuracy referred to the correctness exhibited by the participants when using the triage tool against a set of predetermined profiles, each associated with expected triage codes. During the FSEx exercises, participants were trained using predetermined algorithms (START) and encountered simulated casualties presenting with specific signs and symptoms. Although far from being perfect, START triage is still extensively used in real-world applications and embedded in several local operational plans [17, 28]. The use of the DCCs, which presents vitals in a written format, combined with trained live actors (who walk and follow commands based on their profiles), provides a foundation to confidently assert that strict adherence to the algorithm, along with transparent provision of all necessary information, should yield very high FRs’ triage accuracy. However, we identified a 16.4% inaccuracy, which we hypothesize could be attributed to other clinical factors integrated by clinicians in their triage decision-making. Concerning our primary objective, the multinomial regression model for overtriage identified significant associations with elevated heart rate, higher H-AIS and T-AIS, and a lower overall ISS. Clinicians have traditionally considered heart rate, a parameter notably absent in the START algorithm, as a potential indicator of a more "severe" or shocked patient. It is plausible that the occurrence of tachycardia in patients categorized within a lower triage level may have influenced clinicians to assign a triage code higher than the expected one. Similar considerations apply to the heightened H-AIS and T-AIS. The AIS is an anatomically-based injury severity scoring system. It classifies each injury by body region on a six point scale. Although formal anatomical evaluation or triage wasn't requested from FSEx participants, it was hypothesized that providers' field triage decisions might be indirectly influenced by visible anatomical injuries (e.g., bruises, fractures, penetrating trauma, amputations) or their consequences (e.g., coma, bleeding). Therefore, the AIS score was included in the statistical analysis, as it serves as a proxy for the anatomical injuries sustained. Trauma to the head or chest, such as penetrating or open chest wounds in a walking patient, might have contributed to instances of overtriage. When considering undertriage, independently associated factors are higher systolic blood pressure, better AVPU class scale and higher A-AIS. Indeed, DCCs provided participants in the prehospital setting with information on “advanced” vital parameters (such as blood pressure), typically accessible only through the presence of monitors in a more advanced phase of the MCI response, when more resources arrive on scene or an advanced medical post is established [29]. Blood pressure, which is not “per se” included in the START evaluation, is however widely used in assessing the hemodynamic stability of trauma patients, thus the observation that a higher systolic could be perceived as indicative of a more “stable” patient and as such the link with undertriage is not surprising [30]. Consciousness itself could follow the same approach, very young and fit patients usually tolerate a higher degree of shock without compromising their consciousness level and relying on the consciousness state probably led to some undertriage in our cohort. Finally, in contrast to noticeable signs of head and chest injuries (like changes in consciousness, penetrating objects, or respiratory issues) diagnosing abdominal injuries can be challenging, especially when point-of-care ultrasound is not readily available. This difficulty in clinical diagnosis probably contributes to the higher A-AIS in the undertriage model. On a side note, it is interesting to note that our method of offering additional information on casualties beyond what a typical first responder might gather in their initial assessment of an MCI casualty (e.g., heart rate, systolic blood pressure, blood oxygen saturation) is a forefront of what is likely to occur in the future with the integration of new technologies in MCI response [31, 32]. Indeed, in the evolving landscape of prehospital emergency response, a discernible trend is the increasing integration of sensors and new technologies aimed at enhancing the performance of FRs also by providing additional vitals compared to what is normally available and accessible during MCIs. Similarly to what happened during our study, this influx of additional data has the potential to significantly influence FRs’ clinical assessment, and impact the application of triage protocols, thus potentially affecting the delicate balance between available resources and urgent needs. Hence, there is a pressing need for forthcoming studies to explore these dynamics and consider the potential need to adjust our approach to primary triage in MCIs. Concerning our secondary objective, the AFT model revealed a significant association between the assignment of a triage red code and shorter duration in triage-to-scene departure time. Given that one of the objectives of triage is to prioritize the transportation of individuals across different triage categories, it is unsurprising that patients categorized as red experienced a shorter duration on the scene. Intriguingly, we noted that patients with lower SpO2 spent more time on the scene, potentially indicating that clinicians frequently chose to address airway management at the prehospital scene, thereby prolonging the triage-to-scene departure time. Conversely, patients with higher SpO_2_, requiring no airway and breathing interventions, were evacuated more rapidly. The same consideration can be made for increasing ISS, which could have prompted participants to perform treatment and stabilization maneuvers on site, thus impacting on the time on scene. A reduced time-to-triage departure time was linked to increased provider experience, aligning with the outcomes observed in our previous study wherein “expert” teams demonstrated superior proficiency in both triage and on-scene management [16]. The underlying assumption is that expert teams possess a more comprehensive understanding of prehospital processes and recognize the imperative to swiftly clear the scene, acknowledging the hospital as the definitive care location where casualties can receive optimal treatment. This perspective is consistent with existing literature, which underscores that prolonged prehospital times are correlated with unfavorable hospital outcomes [10].

### Limitations

Several limitations should be acknowledged in this study. Firstly, it is a retrospective analysis of previously collected data. Secondly, despite the efforts to organize the exercises in as realistic a manner as possible, they remain simulations. Consequently, the simulated casualties may only reflect a limited range of changes in their clinical condition, posing challenges to the real-world transferability of the study's findings.

Nevertheless, in the field of disaster medicine, it is common practice to use simulation data as a surrogate to inform further research or propose changes for evaluation in clinical practice. Third, we cannot exclude that the FSEx was not perceived realistic enough from the participants to generate enough stress that could potentially hinder their performance and as such we cannot exclude that real-world performances could be worse (or better) than reported. Fourth, it is worth remembering that ISS and AIS are mainly used for prognostic purposes and their use as anatomical descriptors can be considered at best an approximation. Lastly, due to the inability to identify rescuer-related individual experience, we opted to consider group-level experience as a variable in the data analysis.

## Conclusions

Understanding the predictors influencing triage and scene management decision-making by healthcare professionals responding to a mass casualty may facilitate the development of tailored training pathways regarding triage and scene management. In the primary evaluation of MCI casualties during simulated exercises, altered values of heart rate, blood pressure and AVPU influenced the application of the conventional START algorithm, an observation that suggests the necessity to potentially reevaluate the initial approach to MCI casualties considering the increasing likelihood of immediate access to these parameters through technological advancement. Similarly, mistriage was linked to T-AIS, H-AIS and A-AIS, underscoring the influence of realistic moulage casualties on the application of the START algorithm. This implies that clinicians are inclined to incorporate an anatomical assessment more attentively even during their initial evaluations. Additionally, we noticed an inclination to retain patients with more severe conditions possibly requiring interventions (such as casualties with higher ISS scores or with airway and breathing issues) on the scene for initial stabilization.

## Supplementary Information

**Additional file 1.**

## Data Availability

The data that support the findings of this study are available from the corresponding author upon reasonable request.
