# Supplementary material for: Factors affecting the accuracy of prehospital triage application and prehospital scene time in simulated mass casualty incidents
Source: Scand J Trauma Resusc Emerg Med. 2024 Sep 26;32:97. doi: 10.1186/s13049-024-01257-3 (PMC11426006; doi:10.1186/s13049-024-01257-3)
Supplement: Supplementary file 1 — Additional file 1. [file 13049_2024_1257_MOESM1_ESM.docx]

Factors Affecting the Accuracy of Prehospital Triage Application and Prehospital Scene Time in Simulated Mass Casualty Incidents.

Figure 1S - Example of Dynamic Casualty Card used through the exercises……………………………………………………………….1

Figure 2S - Graphical comparison between non-parametric distribution and Weibull distribution for triage-to-exit time……………….2

Table 1S - Descriptive characteristics of study full scale exercises…………………………………………………………………………..3


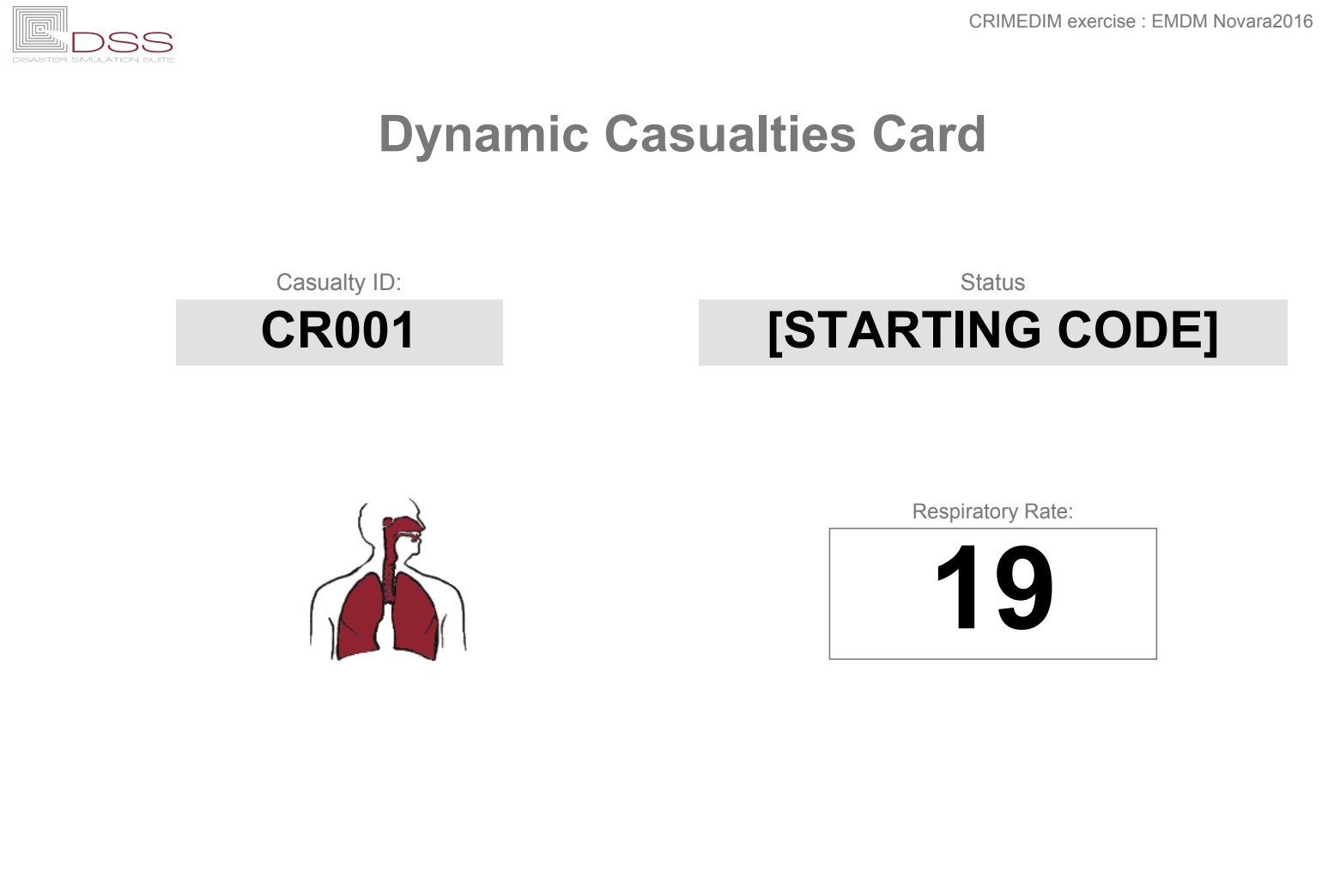

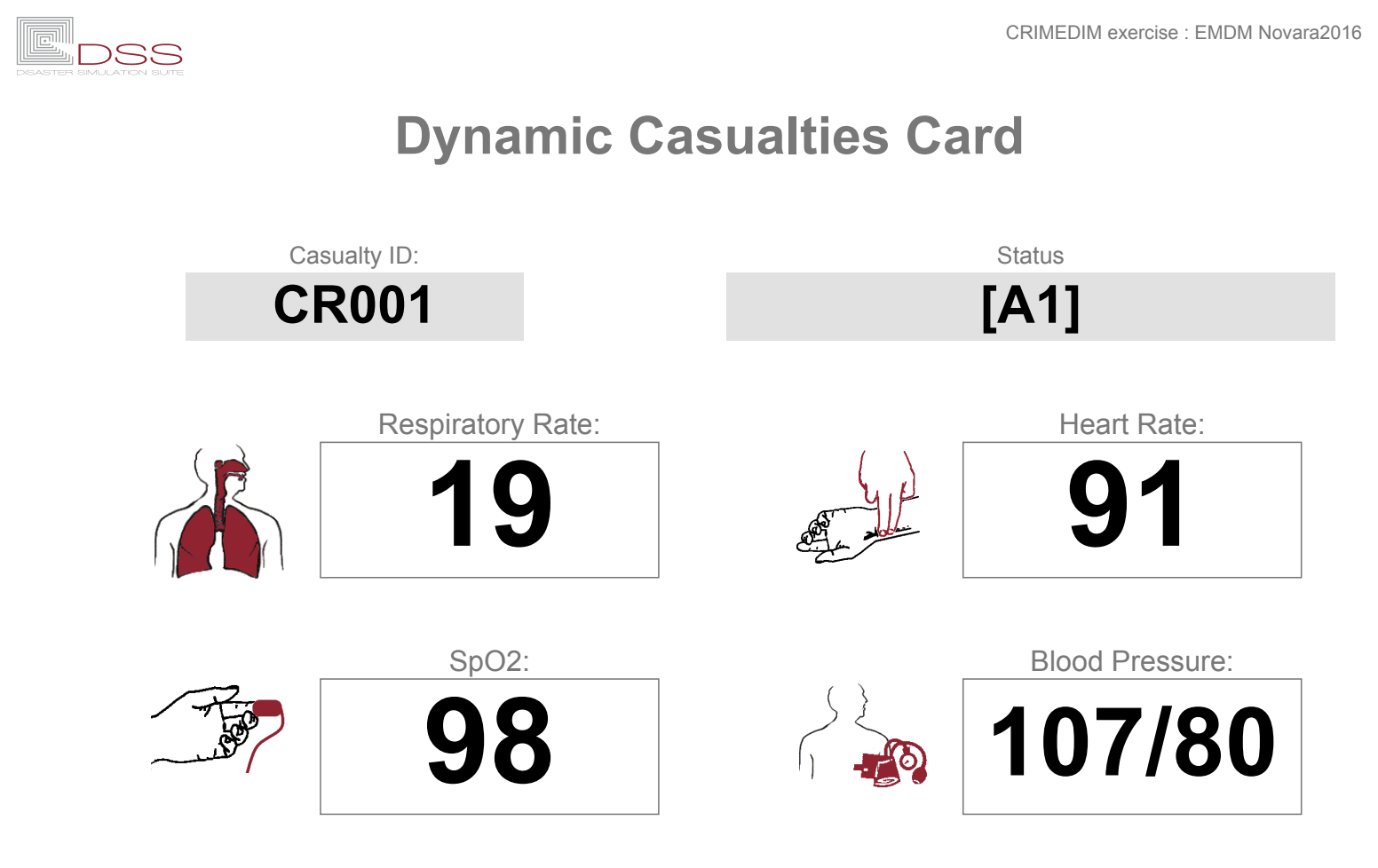


Figure 1S - Example of Dynamic Casualty Card used through the exercises. On the left a starting code card only portraying respiratory rate; on the right a card displaying more vitals, usually displayed after the initial triage.


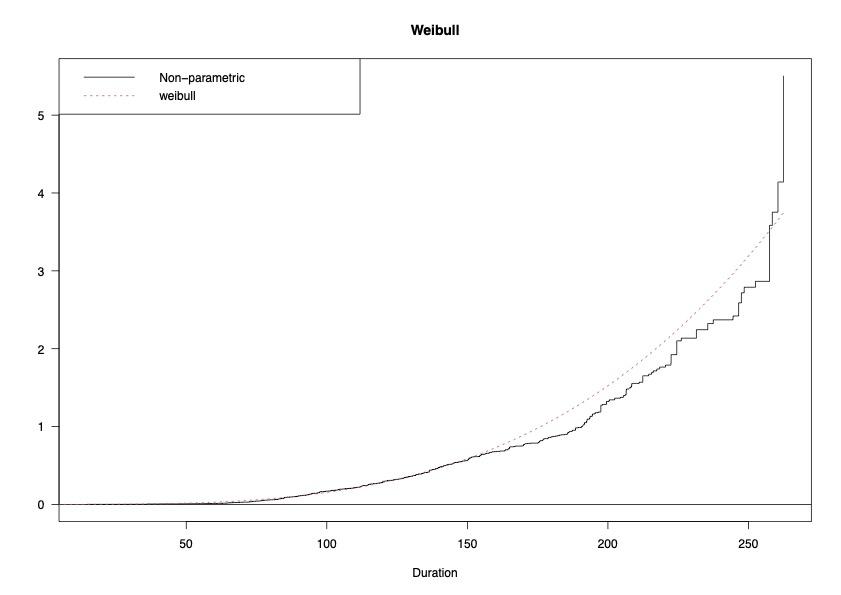


Figure 2S - Graphical comparison between non-parametric distribution and Weibull distribution for triage-to-exit time.

| FSEx name | FSEx date and start time | Scenario | Type | Casualty # | R | Y | G | B |
| --- | --- | --- | --- | --- | --- | --- | --- | --- |
| EMDM 2012 | 5/31/12 20:05 | Indoor Explosion with Building Collapse | E | 107 | 10 | 21 | 70 | 6 |
| EMDM 2013 | 5/23/13 18:48 | Outdoor Explosion at a fuel station | E | 105 | 15 | 19 | 60 | 11 |
| EMDM 2014 | 6/4/14 18:43 | Shipwreck | E | 89 | 11 | 26 | 43 | 9 |
| EMDM 2015 | 5/27/15 19:17 | Plane crash on a refugee camp | E | 121 | 13 | 24 | 81 | 3 |
| EMDM 2016 | 5/18/16 19:03 | Building Collapse during a public event | E | 144 | 15 | 27 | 96 | 6 |
| Brescia | 9/11/16 1:55 | Metro Crash | NE | 80 | 16 | 28 | 30 | 6 |
| Lampedusa 2017 | 5/25/17 14:00 | Migrant boat capsizing | NE | 50 | 8 | 12 | 30 | 0 |
| EMDM 2017 | 5/31/17 19:34 | Multiple Road Traffic Crash | E | 110 | 18 | 27 | 59 | 6 |
| Lampedusa 2018 | 5/24/18 12:57 | Migrant boat capsizing | NE | 85 | 8 | 10 | 66 | 1 |
| EMDM 2018 | 6/6/18 19:40 | Migrants fleeing from war zone: shipwreck and war injuries | E | 82 | 13 | 22 | 43 | 4 |
| Lampedusa 2019 | 5/23/19 14:27 | Multi-site tsunami | NE | 98 | 11 | 23 | 59 | 5 |
| EMDM 2019 | 5/29/19 19:40 | Building Collapse during a party | E | 105 | 14 | 19 | 65 | 7 |
| EMDM 2022 | 5/25/22 19:34 | Multiple Road Traffic Crashes | E | 133 | 17 | 38 | 72 | 6 |
|  |  |  |  | 1309 | 169 | 296 | 774 | 70 |

Table 1S - Descriptive characteristics of study full scale exercises

Abbreviation: Type E - Expert; Type NE - Non Expert (see text for further details); Casualty # - total exercise casualty number; R - Red Casualties; Y - Yellow Casualtyes; G - Green Casualties; B - Black Casualties (all expected triage according to the START system)
